# Behavioural and neurological symptoms accompanied by cellular neuroinflammation in IL-10-deficient mice infected with *Plasmodium chabaudi*

**DOI:** 10.1186/s12936-016-1477-1

**Published:** 2016-08-24

**Authors:** Kyle D. Wilson, Sonja J. Stutz, Lorenzo F. Ochoa, Gustavo A. Valbuena, Petra D. Cravens, Kelly T. Dineley, Gracie Vargas, Robin Stephens

**Affiliations:** 1 Department of Microbiology and Immunology, University of Texas Medical Branch, 301 University Blvd., Galveston, TX 77555 USA; 2 Mitchell Center for Neurodegenerative Diseases, Center for Addiction Research, University of Texas Medical Branch, 301 University Blvd., Galveston, TX 77555 USA; 3 Center for Biomedical Engineering, University of Texas Medical Branch, 301 University Blvd., Galveston, TX 77555 USA; 4 Department of Pathology, University of Texas Medical Branch, 301 University Blvd., Galveston, TX 77555 USA; 5 Department of Internal Medicine, Division of Infectious Diseases, University of Texas Medical Branch, 301 University Blvd., Galveston, TX 77555 USA; 6 Department of Neurology, University of Texas Medical Branch, 301 University Blvd., Galveston, TX 77555 USA; 7 Department of Neuroscience and Cell Biology, University of Texas Medical Branch, 301 University Blvd., Galveston, TX 77555 USA; 8 Institute for Human Infections and Immunity, University of Texas Medical Branch, 301 University Blvd., Galveston, TX 77555 USA

**Keywords:** Malaria, Brain, Monocyte, T cell, Neuroinflammation, Mouse

## Abstract

**Background:**

Cerebral malaria is one of the most severe complications of *Plasmodium falciparum* infection and occurs mostly in young African children. This syndrome results from a combination of high levels of parasitaemia and inflammation. Although parasite sequestration in the brain is a feature of the human syndrome, sequestering strains do not uniformly cause severe malaria, suggesting interplay with other factors. Host genetic factors such as mutations in the promoters of the cytokines IL-10 and TNF are also clearly linked to severe disease. *Plasmodium chabaudi,* a rodent malaria parasite, leads to mild illness in wildtype animals. However, IL-10^−/−^ mice respond to parasite with increased levels of pro-inflammatory cytokines IFN-γ and TNF, leading to lethal disease in the absence of sequestration in the brain. These mice also exhibit cerebral symptoms including gross cerebral oedema and haemorrhage, allowing study of these critical features of disease without the influence of sequestration.

**Methods:**

The neurological consequences of *P. chabaudi* infection were investigated by performing a general behavioural screen (SHIRPA). The immune cell populations found in the brain during infection were also analysed using flow cytometry and confocal microscopy.

**Results:**

IL-10^−/−^ mice suffer significant declines in behavioural and physical capacities during infection compared to wildtype. In addition, grip strength and pain sensitivity were affected, suggestive of neurological involvement. Several immune cell populations were identified in the perfused brain on day 7 post-infection, suggesting that they are tightly adherent to the vascular endothelium, or potentially located within the brain parenchyma. There was an increase in both inflammatory monocyte and resident macrophage (CD11b^hi^, CD45^+^, MHCII^+^, Ly6C^+/−^) numbers in IL-10^−/−^ compared to wildtype animals. In addition, the activation state of all monocytes and microglia (CD11b^int^, CD45^−^, MHC-II^+^) were increased. T cells making IFN-γ were also identified in the brain, but were localized within the vasculature, and not the parenchyma.

**Conclusions:**

These studies demonstrate exacerbated neuroinflammation concurrent with development of behavioural symptoms in *P. chabaudi* infection of IL-10^−/−^ animals.

**Electronic supplementary material:**

The online version of this article (doi:10.1186/s12936-016-1477-1) contains supplementary material, which is available to authorized users.

## Background

With 214 million new cases and 438,000 estimated deaths in 2015, malaria remains one of the highest burdens in preventable and treatable infectious diseases worldwide [1]. Effective anti-malarial drugs often result in cure. However, as many as 10 % of *Plasmodium falciparum* infections worldwide result in severe malaria disease, including severe anaemia, pregnancy-associated malaria and cerebral malaria (CM) [2, 3]. CM correlates with high levels of parasitaemia, inflammation, and severe cerebral oedema [3]. There is little evidence that specific strains of *P. falciparum* are linked to this syndrome, although parasite adhesion in the brain and retina is associated with death [4]. On the other hand, host genetic factors, such as mutations in the promoters of the cytokines IL-10 and tumour necrosis factor (TNF), have been correlated with severe disease [5, 6]. Adding to the complex aetiology of CM, inflammation also promotes parasite sequestration, as demonstrated in murine models of experimental CM [2]. As a result, it is possible that inflammation is the primary precipitating cause of *P. falciparum* sequestration in the human brain and other organs.

*Plasmodium chabaudi*, a rodent malaria species, leads to uncomplicated disease in wildtype (WT) animals. However, in IL-10-deficient (IL-10^−/−^) mice, *P. chabaudi* infection leads to lethal disease characterized by cerebral pathology, including gross cerebral oedema and haemorrhage [7], in the absence of cerebral sequestration [8]. In *P. chabaudi*, sequestration has been documented in several organs, including liver and lungs, but adherent parasite is not readily detectable in the brain vasculature [8, 9]. On the other hand, there is a strong spike of systemic, pro-inflammatory, TNF at the peak of infection that has been shown to cause the lethal pathology in this model [10]. As such, it is clear that inflammatory cytokines can promote, and anti-inflammatory cytokines protect from, malaria-related pathology in murine models [11–13]. In humans, a strong balance of the anti-inflammatory cytokine IL-10 with TNF is also known to be critical for control of parasitaemia and pathology [14].

Histopathological studies from fatal CM cases have provided insight into the pathologic events that correlate with fatal disease [15–17]. Recent striking studies linked evidence of severe cerebral oedema with death [3]. Cytokines such as TNF can cause vascular leakage and oedema; however, the causal mechanisms leading to blood–brain barrier breakdown and brain swelling in human CM cases are not clear. While sequestration is cited as a cause of vascular blockage and oedema [18], this has not been sufficiently demonstrated to date. Fortunately, animal models have been developed that share significant similarities to human cerebral malaria. In *Plasmodium berghei* ANKA-induced experimental cerebral malaria (ECM) infection, pathogenic mononuclear cells accumulate in cerebral blood vessels as a result of inflammatory TNF secretion and the upregulation of chemokine receptors like C-X chemokine receptor 3 (CXCR3) on T cells [19] and cell-adhesion molecules such as intercellular adhesion molecule-1 (ICAM-1) on the vascular endothelium [20]. Furthermore, cytotoxic CD8^+^ T cell recruitment, occurring downstream of IFN-γ, can directly damage microvascular endothelial cells and contribute to oedema [21–24].

In this study, the *P. chabaudi* murine model of malaria in IL-10^−/−^ mice was used. These animals exhibit a severe disease phenotype [25, 26] including signs of neurological involvement [7]. Previous studies in the Langhorne laboratory have shown severe brain oedema, as well as micro- and macro-haemorrhages [7]. However, there is no evidence of significant parasite sequestration in the brain [8, 9]. CD4^+^ IFN-γ^+^ T cells are the primary source of IL-10 in *P. chabaudi* infection [27], and neutralization of TNF ameliorates the severe phenotype, while TGF-β antibodies exacerbates disease [10]. This model provides an opportunity to investigate the effect of inflammatory cytokines, as separate from the effect of parasite sequestration in the brain, on neurological damage during malaria infection.

Neuroinflammation and behavioural changes have not been studied in the lethal course of disease in *P. chabaudi*-infected IL-10^−/−^ animals. Furthermore, the type and location of immune cells in the brain are unknown in this model. Therefore, using the SHIRPA behavioural screen, general health, neurological reflexes, and baseline behaviour were assayed. Multiple deficiencies in these measures were observed in infected IL-10^−/−^ mice compared with even the most moribund WT animals. A significant population of monocytes was found in the brains of *P. chabaudi*-infected IL-10^−/−^ mice by flow cytometry. T cells were also found in the brain, although not at significantly higher levels than WT animals. Interestingly, the infiltration of T cells was localized primarily to blood vessels and not the brain parenchyma. This study supports the role of cellular inflammation in promoting lethal malarial pathology and behavioural changes in mice infected with *P. chabaudi.*

## Methods

### Mice

C57Bl/6 (WT) and B6.129P2-Il10^tm1Cgn^/J (IL-10^−/−^) animals (The Jackson Laboratory, Bar Harbor, ME) were bred in The University of Texas Medical Branch (UTMB) animal care facility. Experimental animals were between 6 and 12 weeks of age at the time of infection. All animals were kept in specific-pathogen free housing with ad libitum access to food and water. General health, neurological, and behavioural analyses were performed on 16 IL-10^−/−^ and 16 wildtype mice (female only) in UTMB’s *Rodent in Vivo Assessment* (*RIVA*) core facility (directed by Dr. Kelly Dineley) housed within the Center for Addiction Research (directed by Dr. Kathryn Cunningham).

### Parasite and infection

Frozen stocks of *Plasmodium chabaudi chabaudi* (*AS*)-infected RBCs (iRBCs) (Jean Langhorne, Francis Crick Institute, London, UK) stored at −80 °C were thawed and injected intraperitoneally (i.p.) into C57Bl/6 mice. Parasitaemia was assessed by preparing thin blood smears stained with Diff-Quik (Siemens Healthcare Diagnostics, Newark, DE) and counted using a light microscope. Parasitized blood was diluted in Krebs glucose, and normal saline to deliver 10^5^ iRBCs in 200 μL i.p. Thin blood smears were collected at regular intervals to monitor for peripheral parasitaemia.

### Animal body temperature and weight

Internal body temperature measurements were determined by subcutaneous implantation of IPTT-300 temperature transponders and read using a DAS-6007 reader system (BMDS, Seaford, DE). Animal weights were measured using an OHAUS CS 200 portable balance (OHAUS, Parsippany, NJ).

### Behavioural studies

Beginning on day three post-infection, throughout the peak of parasitaemia (days 9–11), and up to day 14, all surviving animals were assessed using a modified SmithKline Beecham, Harwell, Imperial College, Royal London Hospital, phenotype assessment (SHIRPA) protocol [28]. This comprehensive behavioural assessment involves a battery of 33 semi-quantitative tests for general health and sensory function, baseline behaviours and neurological reflexes. Higher scores are given for measures showing higher functional ability. The procedures were carried out in an open testing environment away from the home cage, and took 15–20 min per animal daily.

Initially, observation of undisturbed behaviour was conducted with the mouse in an open-bottomed viewing jar placed on top of a metal grid suspended above a piece of white paper for 3 min, during which Body Position, Spontaneous Activity, Respiration Rate, and Tremor were assessed. *Body Position* scores ranged from 0 (completely flat) to 5 (repeated vertical leaping). *Spontaneous Activity* scores ranged from 0 (none) to 4 (rapid/dart movement). *Respiration Rate* scores ranged from 0 (irregular) to 3 (hyperventilation). *Tremor* scores ranged from 0 (important) to 2 (none). At the end of the observation period, the number of *fecal pellets* and *urinary stains* accumulated on the paper was recorded. *Transfer arousal* is measured by transferring the animal in the jar quickly to a gridded arena (3 × 5 grid) from ~6 to 8 inches in the air. The immediate reaction is recorded with scores ranging from 0 (no movement) to 6 [19]. *Locomotor activity* is the number of squares entered by all four feet in 30 s after transfer. *Palpebral closure* is scored from 0 (eyes closed) to 2 (eyes wide open), and *piloerection* is 0 (present) or 1 (absent). *Gait* is observed as the animal traverses the arena and is scored from 0 (incapacity) to 3 (normal). *Pelvic elevation* notes the height the animal’s pelvis as it moves, and is scored from 0 (markedly flattened) to 3 (elevated). Similarly, *tail elevation* scores range from 0 (dragging) to 2 (elevated). *Touch escape* measures the reaction to a finger stroke, and is scored from 0 (no response) to 3 (escape response to approach). *Positional passivity* records the animal’s response to sequential handling, with scores ranging from 0 (no struggle) to 4 (struggles when held by tail). *Trunk curl* is recorded as either 0 (absent) or 1 (present) and *limb grasping* is either 0 (absent) or 1 (present), while holding the animal by the tail. As the animal is lowered back down to the wire grid, *visual placing* (how early the animal reaches out for the grid) is scored from 0 (none) to 4 (early extension at 25 mm). As the animal grips the grid, a gentle horizontal pressure is applied to assess qualitative *grip strength*, scored from 0 (none) to 4 (unusually strong). *Body tone* is assessed by compressing the sides of the animal between the thumb and index finger, and scored from 0 (flaccid) to 2 (extreme resistance). *Pinna reflex* is observed by ear retraction to the tip of a fine cotton swab, recorded as 0 (none) to 2 (repetitive flick). *Corneal reflex* is measured by lightly touching the cornea of the animal with a cotton swab and scored from 0 (none) to 2 (multiple eye blinks). *Toe pinch* is assessed by gentle compression of a hind foot digit with fine forceps to observe the response, scored from 0 (none) to 4 (repeated extension and flexion). *Wire maneuver* is assessed by measuring the length of grasp on an inverted wire grid, scored from 0 (falls immediately) to 4 (active grip with hind legs). *Skin color* 0 (blanched) to 2 (flushed); *heart rate* 0 (slow) to 2 (fast); *limb tone* as measured by resistance to gentle fingertip pressure of the plantar surface of the hind paw, scored 0 (no resistance) to 4 (extreme resistance); *abdominal tone* by palpation of the abdomen, scored 0 (flaccid) to 2 (extreme resistance); *lacrimation* as 0 (present) or 1 (absent). A small dowel is inserted between the teeth at the side of the animal’s mouth to measure *salivation*, recorded as 0 (wet zone entire sub-maxillary area) to 2 (none), and *provoked biting*, recorded as 0 (present) or 1 (absent). *Righting reflex* is scored from an upside-down position near the surface and observing the responding effort to upright itself upon release, scored from 0 (fails to right) to 3 (lands on feet). *Contact righting reflex* is assessed by placing the animal inside a plastic restrainer tube and inverting it to assess its ability to sense its position and correct, scored as 0 (absent) or 1 (present). Throughout this entire battery of tests, *vocalization, urination, and general fear, irritability, or aggression* were recorded as either being 0 (present) or 1 (absent). For analysis, SHIRPA data was pooled from infected IL-10^−/−^ mice exhibiting fatal symptoms early during the acute phase of infection (<12 days post-inoculation) and synchronized to the time of death of the individual mouse. Synchronized IL-10^−/−^ SHIRPA scores were compared to the peak of infection for C57Bl/6J control animals (day 10 post-inoculation).

### Forelimb grip strength

Quantitative forelimb *grip strength* was measured using a Chatillon DFIS-2 digital force gauge (Ametek, Largo, FL) measuring maximal grams of resistance by restraining the animal at the base of the tail and allowing it to grasp the tension bar whilst pulling back gently until the animal released its grip. Resistance was calculated in real-time and the maximal resistance achieved by each mouse was averaged. Animals were subjected to three trials (with at least 10 min of rest between each trial).

### Tail flick analgesia

To test for nociceptive *pain sensitivity*, animals were placed on a tail flick analgesia meter (Columbus Instruments, Columbus, OH) and gently restrained. Latency to tail flick was recorded as the time (in seconds) required for the mouse to move its tail out of the path of a heat source. Animals were subjected to three trials with no less than 20 min between trials.

### Flow cytometry

Mice infected with *Plasmodium chabaudi* were anesthetized with inhaled isoflurane (0.25–3 %, to effect) or ketamine/dexmedetomidine cocktail (60–75 mg/kg K, 0.5–1.0 mg/kg D) i.p. and perfused with 20 mLs PBS via cardiac puncture. Single-cell suspensions from brains were made in PBS by pressing through a 70 µm cell strainer, enriched within a 30/70 % Percoll interface (Sigma-Aldrich, St. Louis, MO), and stained in PBS supplemented with 2 % FBS (Sigma-Aldrich) and 0.1 % sodium azide with anti-CD16/32 (2.4G2) supernatant (BioXCell). This was followed by combinations of FITC, PE, PerCP-Cy5.5, PE/cyanine 7 (Cy7), PE/Cy5, (allophycocyanin)-, or allophycocyanin/eflour780–conjugated Abs (all from eBioscience); CD4-Brilliant Violet 785 (BioLegend, San Diego, CA); Ly5.2 PE, Ly-6C FITC, TNF-PE/Cy7 (all from eBioscience), and CD11b-Biotin, followed by Streptavidin-PE/Cy5. Cells were collected on a LSRII Fortessa using FACSDiva software (BD Biosciences, San Jose, CA) and analysed in FlowJo (version 9.7, TreeStar, Ashland, OR). Cells were analysed within 1 h of staining. For intracellular staining of IFNγ and TNF, cells were stimulated for 3 h with PMA/Ionomycin and Brefeldin A for 2 additional hours before fixation in 2 % paraformaldehyde (Sigma-Aldrich) and permeabilization using BD Perm/Wash buffer. Subsequently, cells were incubated with IFNγ-Brilliant Violet 605 (BioLegend) and TNF PE/Cy7 (eBioscience) for 40 min at 4 °C and washed. Compensation was performed in FlowJo using single-stained splenocytes (using CD4 in all colors). For presentation, data from three to four mice were concatenated to achieve sufficient cell numbers, from which averages and SEM were calculated.

### Confocal microscopy

Infected IL-10^−/−^ and WT animals were injected with 2 × 10^6^ Cell Trace Violet^+^ (CTV^+^) CD4 T cells i.p. 3.5 h before sacrifice, and 40ug of DyLight488 labelled *Lycopersicon esculentum* (Tomato) Lectin i.v. 20 min before sacrifice without intracardiac perfusion. After 48 h of post-fixation in 4 % paraformaldehyde and 48 h of cryo-protection in 30 % sucrose, 30 μm frozen sagittal sections of mouse brains were made using Tissue Freezing Medium (Triangle Biomedical Sciences, Durham, NC), mounted on glass slides with fluorescent mounting medium (DAKO, Carpinteria, CA), and cover slipped. Confocal images were acquired on a Fluoview 1000MPE system configured with an upright BX61 microscope (Olympus, Center Valley, PA). Images were analysed using Olympus Fluoview FV1000-ASW 2.0 Viewer.

### Statistics

Where indicated, experiments were analysed by one-way ANOVA, followed by Student’s *t* test or Wilcoxon rank-sum test for nonparametric data, in Prism (GraphPad, La Jolla, CA) and SigmaPlot 12.0 (Systat Software, San Jose, CA): *p ≤ 0.05, **p ≤ 0.01, ***p ≤ 0.001. Error bars represent ± SEM.

## Results

### IL-10^−/−^ animals infected with *P. chabaudi* exhibit deficits in behavioural tests prior to death

Infected IL-10^−/−^ mice suffered a more severe course of disease with decreased survival (females: 6/39, 15.4 %; males: 9/20, 45.0 %; Additional file 1) and significant weight and temperature loss, despite comparable parasitaemia to WT animals (Additional file 1). The majority of IL-10^−/−^ animals succumbed between days 7 and 11 post-infection. This is comparable to previous studies in *P. chabaudi*, although mortality is increased [25, 26]. While severe anaemia is an important cause of death in *P. chabaudi* infection of several strains of mice [29, 30], anaemia peaks in both IL-10^−/−^ and WT mice at day 10 post-infection [25], and not during the time frame when most IL-10^−/−^ animals are succumbing to infection (days 7–9 post-infection, Additional file 1). Furthermore, anaemia is not an accurate predictor of mortality [10], nor is it correlated with percent parasitaemia in this model [31]. In order to determine the behavioural phenotype of IL-10^−/−^ mice infected with *P. chabaudi*, IL-10^−/−^ and WT mice were infected with 10^5^*P. chabaudi*-infected red blood cells (iRBCs), and a comprehensive animal health and behaviour assessment was performed daily. The SHIRPA, a rigorous and semi-quantitative battery of tests, has revealed significant deficits in animals with *P. berghei* ANKA-induced experimental cerebral malaria [32]. During the acute phase of infection (5–14 days post-infection), IL-10^−/−^ mice demonstrated significant deficiencies in many behavioural tests when compared to infection-matched WT mice. However, more results of the SHIRPA assessment became significant when analysed at time points closer to death, even when compared to WT mice at their most severe state (10 days post-infection). This analysis is shown in Table 1. Deficiencies in each SHIRPA functional domain in infected IL-10^−/−^ mice were found, suggesting that malaria infection affects a broad array of neurological functions.

**Table 1:** *Plasmodium chabaudi*-infected IL-10^−/−^ mice show impaired performance in the SHIRPA assessment (Categories adapted from [32])

	Day 10	IL-10^−/−^ vs. C57BL/6J									
	C57BL/6J	<120 h		<96 h		<72 h		<48 h		<24 h	
	n = 16	n = 4	p	n = 6	P	n = 5	p	n = 5	p	n = 8	p
Weight	17.8 (1.6)	18.2 (2.7)	NS	17.4 (1.9)	NS	16.8 (3.1)	NS	16.4 (2.3)	NS	14.7 (1.9)	<0.001*
Reflex/sensory function											
Visual placing	4 (4/4)	3.5 (3/4)	NS	3 (3/4)	0.015*	3 (3/4)	0.039*	3 (3/3.5)	0.005*	3 (3/4)	0.006*
Pinna reflex	1 (1/1)	1 (1/1)	NS	1 (1/1)	NS	1 (1/1)	NS	1 (1/1)	NS	1 (1/1)	NS
Corneal reflex	1 (1/1)	1 (1/1)	NS	1 (1/1)	NS	1 (1/1)	NS	1 (1/1)	NS	1 (1/1)	NS
Toe pinch	4 (4/4)	4 (4/4)	NS	4 (4/4)	NS	4 (4/4)	NS	4 (4/4)	NS	4 (4/4)	NS
Righting reflex	3 (3/3)	3 (3/3)	NS	3 (3/3)	NS	3 (3/3)	NS	3 (2.5/3)	NS	3 (1/3)	0.018*
Cont. right. reflex	1 (1/1)	1 (1/1)	NS	1 (1/1)	NS	1 (1/1)	NS	1 (1/1)	NS	1 (1/1)	NS
Neuropsychiatric state											
Spont. activity	2 (2/2)	2 (2/2)	NS	2 (2/2)	NS	2 (1/2)	0.012*	1 (1/2)	0.001*	1 (1/1.5)	<0.001*
Transfer arousal	5 (5/5)	5 (4.25/5)	NS	5 (4/5)	NS	5 (4.5/5)	NS	3 (2.5/5)	0.017*	2 (2/4.5)	<0.001*
Touch escape	2 (2/2)	2 (2/2)	NS	2 (2/2)	NS	2 (1.5/2)	NS	2 (2/2)	NS	2 (1/2)	0.005*
Posit. passivity	4 (4/4)	4 (4/4)	NS	4 (4/4)	NS	4 (4/4)	NS	4 (4/4)	NS	4 (4/4)	NS
Provoked biting	0 (0/0)	0 (0/0)	NS	0 (0/0)	NS	0 (0/0)	NS	0 (0/0.5)	NS	1 (0/1)	0.001*
Fear	1 (1/1)	1 (1/1)	NS	1 (1/1)	NS	1 (1/1)	NS	0 (0/1)	0.001*	0 (0/1)	<0.001*
Irritability	1 (1/1)	1 (1/1)	NS	1 (1/1)	NS	1 (1/1)	NS	1 (1/1)	NS	1 (0.5/1)	NS
Aggression	1 (1/1)	1 (1/1)	NS	1 (1/1)	NS	1 (1/1)	NS	1 (1/1)	NS	1 (1/1)	NS
Vocalization	1 (1/1)	1 (1/1)	NS	1 (1/1)	NS	1 (1/1)	NS	1 (0/1)	NS	0 (0/0.5)	0.002*
Motor behavior											
Body position	4 (3/4)	4 (4/4)	NS	4 (4/4)	NS	3 (3/3.5)	NS	3 (3/3.5)	NS	3 (3/3.5)	0.043*
Tremor	2 (2/2)	2 (2/2)	NS	2 (2/2)	NS	2 (2/2)	NS	2 (1/2)	0.012*	2 (1/2)	0.005*
Locom. activity	6.2 (4.9)	8.8 (5.4)	NS	14.3 (8.2)	0.009*	8.6 (4.8)	NS	4.2 (2.8)	NS	3.7 (1.9)	NS
Pelvic elevation	2 (2/2)	2 (2/2)	NS	2 (2/2)	NS	2 (2/2)	NS	2 (2/2)	NS	2 (1.5/2)	NS
Gait	3 (3/3)	3 (3/3)	NS	3 (3/3)	NS	3 (3/3)	NS	3 (2/3)	0.012*	3 (1.5/3)	0.005*
Tail elevation	1 (1/1)	1 (1/1)	NS	1 (1/1)	NS	1 (1/1)	NS	1 (1/1)	NS	1 (0/1)	0.018*
Trunk curl	1 (1/1)	1 (1/1)	NS	1 (1/1)	NS	1 (1/1)	NS	1 (1/1)	NS	1 (1/1)	NS
Limb grasping	1 (1/1)	1 (1/1)	NS	1 (1/1)	NS	1 (1/1)	NS	1 (1/1)	NS	1 (1/1)	NS
Wire maneuver	4 (4/4)	4 (4/4)	NS	4 (4/4)	NS	4 (4/4)	NS	4 (4/4)	NS	4 (2/4)	0.018*
Negative geotaxis	4 (4/4)	4 (4/4)	NS	4 (4/4)	NS	4 (4/4)	NS	4 (4/4)	NS	4 (0/4)	0.018*
Autonomous function											
Resp. rate	3 (2/3)	2 (2/2)	0.034*	2 (2/2)	0.012*	3 (2/3)	NS	2 (2/3)	NS	2 (2/3)	NS
Feces	1.3 (1.6)	2.0 (0.8)	NS	1.0 (1.5)	NS	0.2 (0.4)	NS	0.4 (0.5)	NS	0.4 (1.0)	NS
Urine	0 (0/0)	0 (0/0)	NS	0 (0/0)	NS	0 (0/0)	NS	0 (0/0)	NS	0 (0/0)	NS
Palpebral closure	2 (2/2)	2 (2/2)	NS	2 (2/2)	NS	2 (1.5/2)	NS	2 (1/2)	0.012*	2 (1/2)	0.005*
Piloerection	0 (0/0.75)	1 (1/1)	0.009*	1 (1/1)	0.002*	1 (0/1)	NS	0 (0/1)	NS	0 (0/1)	NS
Skin color	1 (1/1)	1 (1/1)	NS	1 (1/1)	NS	1 (1/1)	NS	1 (1/1)	NS	1 (0/1)	NS
Heart rate	2 (1.25/2)	1 (1/1)	0.009*	1 (1/1)	0.002*	1 (1/2)	NS	1 (1/1.5)	0.035*	1 (1/1)	0.002*
Lacrimation	1 (1/1)	1 (1/1)	NS	1 (1/1)	NS	1 (0.5/1)	NS	1 (0/1)	0.012*	1 (0/1)	0.005*
Salivation	1 (0/2)	0 (0/0.75)	NS	0.5 (0/1)	NS	1 (0/2)	NS	1 (0/2)	NS	2 (2/2)	0.015*
Temperature	36.4 (0.9)	38.5 (0.2)	0.019*	38.7 (0.3)	0.011*	37.9 (0.3)	0.031*	35.5 (3.1)	NS	33.4 (2.9)	0.021*
Muscle tone and strength											
Grip strength	3 (3/3)	3 (3/3)	NS	3 (3/3)	NS	3 (3/3)	NS	3 (2.5/3)	NS	2 (1.5/3)	<0.001*
Body tone	1 (1/1)	1 (1/1)	NS	1 (1/1)	NS	1 (1/1)	NS	1 (1/1)	NS	1 (0.5/1)	NS
Limb tone	2 (2/2)	2 (2/2.75)	NS	2 (2/3)	NS	2 (2/3)	NS	2 (2/3)	NS	2 (2/2)	NS
Abdominal tone	1 (1/1)	1 (1/1)	NS	1 (1/1)	NS	1 (1/1)	NS	1 (1/1)	NS	1 (1/1)	NS

Compiled from two independent experiments, female IL-10^−/−^ mice (n = 11) and C57Bl/6 J mice (n = 16) were inoculated with 10^5^ Pcc-iRBCs i.p. and followed for 10–14 days during the acute phase of infection in which the SHIRPA comprehensive behavioral assessment was performed daily. SHIRPA data was pooled from infected IL-10^−/−^ mice exhibiting fatal symptoms early during the acute phase of infection (<12 days post-inoculation) and synchronized to the time of death of the individual mouse. Synchronized IL-10^−/−^ SHIRPA scores were compared to the peak of infection for C57Bl/6J control animals (day 10 post-inoculation). Data shown are median (lower/upper quartile) or mean (± SD) where appropriate. Statistical significance determined by Rank Sum Test or Student’s t test (p < 0.05). Significant values are starred and displayed in italic font

**Fig. 1:**
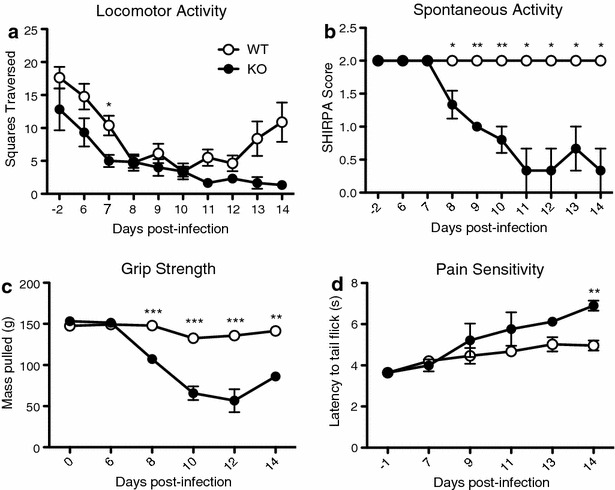
IL-10^−/−^ mice exhibit impaired general health, neurological reflexes and baseline behavioural measures during *Plasmodium chabaudi* infection. As part of the comprehensive SHIRPA behavioural assessment in infection-matched IL-10^−/−^ (KO) and C57Bl/6J (WT) control mice, (**a**) locomotor activity was measured by allowing the mice to explore a novel grid enclosure for 30 s. The number of squares traversed by all four feet was measured within the allotted time. **b** Spontaneous activity was measured by observing the mouse within an enclosed environment for 2 min. Scores ranged from 0 (no activity, resting) to 4 (extremely vigorous, rapid/dart movement), with the baseline score being 2 (vigorous scratch, groom, moderate movement). **c** Animal forelimb grip strength was tested using a digital force-gauging apparatus measuring maximal grams of resistance. Animals were subjected to 3 trials (with at least 10 min of rest between each trial). **d** Nociceptive pain sensitivity was tested by a tail flick analgesia apparatus to measure the latency of tail removal from a nociceptive stimulus. Student’s t test (**a**, **c**, **d**) or Rank sum test (**b**) *p < 0.05, **p < 0.01, ***p < 0.001. *Data points* and *error bars* represent mean values and ± SEM, respectively

Locomotor activity is measured as the number of squares on a grid traversed by mice exploring a new environment, and has been used as a surrogate measure of non-specific sickness behaviour in *P. berghei* ANKA infection [33]. This activity decreased similarly between groups, until day 7 post-infection, from which time on there was a non-significant trend for surviving IL-10^−/−^ animals to move less (Fig. 1a). A decrease in spontaneous activity was specific to the IL-10^−/−^ group, and was reduced beginning 8 days post-infection (Fig. 1b). Furthermore, forelimb grip strength and nociceptive pain sensitivity, measures of motor and pain-sensing neural activity, in infected IL-10^−/−^ mice became significantly different from WT mice on days 8 and 14 post-infection, respectively (Figs. 1c, d). In summary, IL-10^−/−^ mice infected with *P. chabaudi* develop behavioural and functional deficiencies across all categories of the SHIRPA assessment, suggesting damage across several brain areas, including the circuitry underlying locomotor activity, strength, and analgesia.

### IL-10^−/−^ animals show increased CD11b^+^ immune cells and increased microglia activation in the brain during *P. chabaudi* infection

**Fig. 2:**
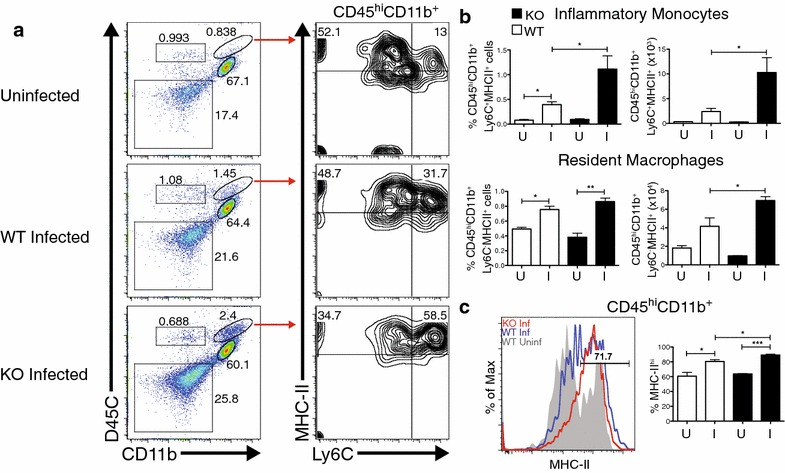
Brains from IL-10^−/−^ animals show increased CD11b^+^ immune cell trafficking and MHC-II upregulation during *Plasmodium chabaudi* infection. Compiled from multiple independent experiments, cells were isolated from PBS-perfused brains of female IL-10^−/−^ (KO) and C57Bl/6J (WT) mice 7–9 days post-infection (**a**) Representative FACS plots showing CD11b^+^CD45^hi^ immune cells in the brain expressing Ly6C and MHC-II. **b** Total percent and number of Ly6C^−^MHC-II^+^ tissue macrophages and of Ly6C^+^MHC-II^+^ inflammatory monocytes within the CD11b^+^CD45^hi^ gate. **c** Histogram and % positive proportion of MHC-II expression and on CD45^hi^CD11b^+^ immune cells. *Histogram colors* Uninfected WT (*Grey*); day 7 and day 9 post-infection WT (*Blue*); day 7–9 post-infection IL-10^−/−^ (*Red*). *I* infected, *U* uninfected. Student’s t test *p < 0.05, **p < 0.01, ***p < 0.001

**Fig. 3:**
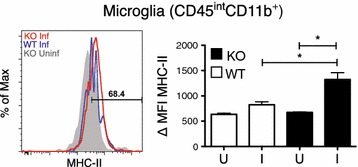
Brains from IL-10^−/−^ animals show increased microglia activation during *Plasmodium chabaudi* infection. Compiled from multiple independent experiments, cells were isolated from PBS-perfused brains of female IL-10^−/−^ (KO) and C57Bl/6J (WT) mice 7–9 days post-infection. Mean fluorescent intensity (MFI) and histogram of MHC-II expression on resident microglia cells (CD11b^+^CD45^int^). *Histogram colors* Uninfected WT (*Grey*); day 7 and day 9 post-infection WT (*Blue*); day 7–9 post-infection IL-10^−/−^ (*Red*). *I* infected, *U* uninfected. Student’s t test *p < 0.05, **p < 0.01

In order to identify the cellular populations present in the brains of IL-10^−/−^ mice infected with *P. chabaudi,* single-cell suspensions from perfused brains were prepared for flow cytometry. During the acute phase of infection (7–9 days post-infection), lymphocytes (CD45^hi^, CD11b^−^), monocytes/macrophages (CD45^hi^, CD11b^+^), microglia (CD45^int^, CD11b^+^), and non-hematopoietic cells (CD45^−^, CD11b^−^) were detected. All infected animals had a significant increase in the fraction of cells in the monocyte/macrophage gate, with the IL-10^−/−^ group increasing the most (Fig. 2a). Within the monocyte/macrophage gate, migrating “inflammatory monocytes” (CD11b^+^, Ly6C^+^, MHC-II^+^) were distinguished from the rest of the macrophage population. Migratory monocytes were increased in the brains of infected IL-10^−/−^ animals (Fig. 2b). CD11b^+^Ly6C^−^MHC-II^+^ macrophages, which include resident and some migratory macrophages, were also increased in both infected WT and IL-10^−/−^ groups. However, the numbers of Ly6C^−^ macrophages in brains of infected IL-10^−/−^ animals were greater compared to infection-matched WT animals and uninfected controls. Furthermore, MHC-II expression was found to be upregulated on the entire CD45^hi^CD11b^+^ monocyte/macrophage population compared to WT infected and uninfected controls, suggesting activation (Fig. 2c). In addition, microglia (CD45^int^, CD11b^+^) in the brains of infected IL-10^−/−^ mice also showed increased expression of MHC-II (Fig. 3). An increase in peripheral immune cell populations and the activation state of resident microglia suggests cellular infiltration in the brains of IL-10^−/−^ mice infected with *P. chabaudi.*

### Mice have IFN-γ-producing T cells in the brain during *P. chabaudi* infection

**Fig. 4:**
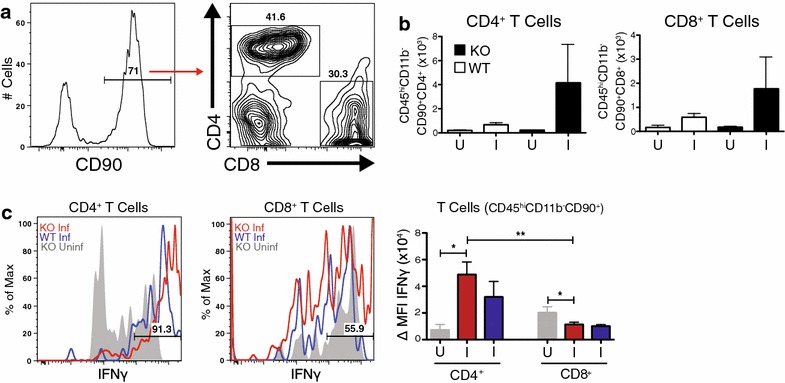
Mice have IFN-γ-producing T cells in the brain during *Plasmodium chabaudi* infection. Compiled from two independent experiments, cells were isolated from PBS-perfused brains of female IL-10^−/−^ (KO) and C57Bl/6J (WT) mice 7–9 days post-infection. **a** Representative FACS plots showing CD11b^−^CD45^hi^ immune cells in the brain, gated on the Thy1.2^+^ population, then CD4 and CD8a. **b** Total cell numbers of CD4 and CD8 T cells within the CD11b^−^CD45^hi^/Thy1.2^+^/CD4^+^ or CD11b^−^CD45^hi^/Thy1.2^+^/CD8^+^ gate, respectively. **c** Histograms showing the expression of IFN-y in CD4^+^ and CD8^+^ T cells. *Right panel* MFI of IFN-y-expressing T cells in infected mice and uninfected controls. Percent positive proportion of infected IL-10^−/−^ mice shown. *Histogram colors* Uninfected IL-10^−/−^ (*Grey*); day 7 and day 9 post-infection WT (*Blue*); day 7–9 post-infection IL-10^−/−^ (*Red*). *I* infected, *U* uninfected

As T cells have been implicated in the pathogenesis of ECM [34–36], and the death of *P. chabaudi*-infected IL-10^−/−^ mice [27], infiltrating CD4^+^ and CD8^+^ T cells were specifically stained within the lymphocyte gate (CD45^hi^, CD11b^−^, Thy1 (CD90)^+^) (Fig. 4a). Staining B cells with CD19 identified a minority of animals with B cells in the brain as well (data not shown). Some IL-10^−/−^ animals had an increased number of CD4^+^ and CD8^+^ T cells in the brain, however it was not found to be significantly different from WT or uninfected controls (Fig. 4b). Almost all of the T cells present within the brains of infected WT and IL-10^−/−^ mice expressed IFN-γ. Importantly, CD4^+^ T cells in IL-10^−/−^ mice expressed much higher levels of IFN-γ per cell than CD8^+^ T cells (Fig. 4c). As the brains were perfused, these cells could be adherent to the vascular endothelium or infiltrating the brain parenchyma. Therefore, it is important to define their localization to determine the function that these cells may play in the pathogenesis of severe malaria.

### CD4^+^ T cells exhibit intravascular localization in IL-10^−/−^ animals infected with *Plasmodium chabaudi*

**Fig. 5:**
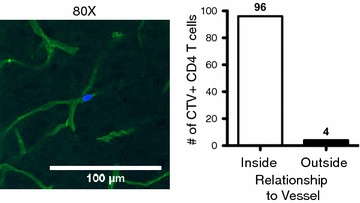
CD4^+^ T cells exhibit intravascular localization in IL-10^−/−^ animals infected with *P. chabaudi*. A 30 μm sagittal section of a female IL-10^−/−^ (KO) brain 8 days post-inoculation with 10^5^ Pcc-iRBCs i.p. Animals were injected with 2 × 10^6^ CTV^+^ CD4 T cells i.p. 3.5 h before sacrifice, and 40 μg of DyLight488 labelled *Lycopersicon esculentum* (Tomato) Lectin i.v. 20 min before sacrifice without perfusion. *Left panel* Confocal image of an adoptively transferred CTV^+^ CD4^+^ T cell located in the Lectin^+^ microvasculature of the KO brain. *Right panel* Quantitation of adoptively transferred CTV^+^ CD4 T cells found inside or outside Lectin^+^ blood vessels within the KO brain. Imaging performed using a 20× objective with 4× digital zoom to visualize individual T cells within microvasculature

To investigate the localization of potentially pathogenic CD4^+^ T cells relative to the vasculature, CellTrace Violet^+^ (CTV^+^) polyclonal CD4^+^ T cells were transferred from infection-matched (day 7 post-infection) IL-10^−/−^ donors, and injected with Tomato lectin, which stains glycoproteins localized to the vascular endothelium. Adoptively transferred cells were visualized by confocal microscopy. The vast majority of T cells remained within the brain vasculature, as opposed to invading the parenchyma (Fig. 5). Upon closer inspection, the T cells that appeared to localize to the parenchyma displayed several anomalies (multiple fluorescent staining, out of focus) that decrease their likelihood of representing true transendothelial migration. Thicker brain sections were also observed with multiphoton microscopy, confirming localization of CD4^+^ T cells in the vasculature (data not shown). In conclusion, CD4^+^ T cells that are responsible for lethal pathology in *P. chabaudi*-infected IL-10^−/−^ mice do not infiltrate the parenchyma, but remain within the blood vessels supplying the brain.

## Discussion

In this study, behavioural and immunological outcomes of *P. chabaudi* infection in IL-10^−/−^ animals were assessed. The goal was to identify the cellular immune populations associated with the neurological syndrome and the fatal phenotype [10, 25, 26]. While brain pathology has been well-documented in this model [10], behavioural assessments have not been performed to confirm that infection had severe effects on general health, neurological reflexes, and baseline behaviours in IL-10^−/−^ mice infected with *P. chabaudi*. Trafficking immune cell populations found in the brain also had not been investigated by flow cytometry or confocal microscopy.

Behavioural studies in the *P. berghei* ANKA model have established a correlation between the deterioration in motor skills and neuromuscular function with histopathological changes associated with ECM [32, 33, 37]. Mice infected with *P. berghei* ANKA and evaluated with the SHIRPA exam showed early indications of neurological consequences via impairment in reflex and sensory functions, along with neuropsychiatric state [32]. As the disease progressed, multiple parameters became significantly different in mice suffering from experimental cerebral malaria versus non-cerebral control mice, which also correlated with mean size of brain haemorrhage [32]. In the present study of animal behaviour during *P. chabaudi* infection of IL-10^−/−^ mice, an association of decreased function with fatal disease was observed. The main finding here was that at the peak of illness, wildtype animals performed better on virtually all behavioural tests than the IL-10^−/−^ mice 24 h prior to death. Thus, deficiencies in functional behaviour domains may serve as early indicators of severe disease as some of these categories were decreased even earlier than 48 h prior to death (Table 1). The behavioural findings in this study are very similar to findings in *P. berghei* ANKA infection [32], suggesting brain involvement in the lethal pathology of *P. chabaudi*-infected IL-10^−/−^ mice. Using this model, Brugat et al. have also shown indications of pathology in the liver and lungs, with concomitant sequestration in these organs. In addition, kidney pathology is present, however, there is no evidence of sequestration in the kidney, suggesting sequestration is not always the primary cause of organ pathology in this model [8]. Infected IL-10^−/−^ mice show an increase in diagnostic markers of kidney damage and leukocyte trafficking to the lungs. These indications are seen in cases of multiple organ failure caused by severe malaria infection [38–40]. Liver failure is also observed in infection of the susceptible mouse strain DBA/2 [41], which is mirrored in BALB/c mice lacking expression of haemoxygenase-1 (HO-1, Hmox1^−/−^) [42], a free haem-catalyzing enzyme under the regulation of IL-10 that has been shown to be important in the pathogenesis of ECM and human CM [43, 44]. The role HO-1 plays in the pathogenesis of severe malaria in IL-10^−/−^ mice infected with *P. chabaudi* has yet to be studied. While there are similarities between this model and ECM, the exact cause of death in IL-10^−/−^ mice is unclear. Therefore, it is important that these findings demonstrate a significant peripheral immune infiltration of the central nervous system (CNS) vasculature, correlating with neurological and behavioural deficits. Given the lack of correlation of sequestration with all organ damage, and given that the mortality of infected IL-10^−/−^ mice can be prevented with neutralization of TNF [10, 41], symptoms in this model are likely attributable to inflammation versus other features downstream of parasite biomass.

*Plasmodium chabaudi* infection in IL-10^−/−^ mice results in vascular leakage and haemorrhage [7], which is also observed in *P. berghei* ANKA infection [45]. This pathological finding has been linked to the accumulation of cytotoxic CD8^+^ T cells in the cerebral microvasculature [21, 23]. The presence of microvascular haemorrhages and cerebral oedema in human CM cases is correlated with adverse outcomes and death [3, 4]. The trafficking of pathogenic T cells and their involvement in mediating ECM is pertinent in our model, as it has been shown that selective deletion of IL-10 from T cells recapitulates the fatal phenotype observed in IL-10^−/−^ mice [27]. However, it is not known at what anatomical sites these cells are lethal. Non-Treg IFN-γ^+^ CD4^+^ T cells were identified as the primary producers of IL-10 in that study, as well as in lethal *P. yoelii* infection [46].

In order to characterize the cellular infiltrate, cell populations in the perfused brain tissue of *P. chabaudi*-infected IL-10^−/−^ mice were identified by flow cytometry. Significant increases in macrophages (CD45^+^CD11b^hi^) of two distinct types were observed in IL-10^−/−^ animals compared to infection-matched WT animals. Inflammatory monocytes (CD11b^+^Ly6C^+^) were identified as well as tissue-resident (CD11b^+^Ly6C^−^) macrophages, though these are also likely to include maturing infiltrating cells [47]. A significant increase in activation of microglia was also measured, suggesting that either cytokines, or contact with other activated cell types, are affecting glial cells, which are an integral part of the brain parenchyma and act as the first line of defense in the CNS.

In this study, CD4^+^ T cells were found in perfused brains, suggesting that they are either adherent or have infiltrated into the brain parenchyma. We further show by confocal microscopy that they are located within the brain vasculature of infected IL-10^−/−^ mice. T cells in the brain were also shown to make IFN-γ, clearly demonstrating that pathologic lymphocytes traffic to the brain vasculature where they could contribute to the decrease in functional behaviour witnessed in IL-10^−/−^ mice infected with *P. chabaudi*. Furthermore, this suggests that infiltration of pathological immune cells may not be necessary to affect neurological function. Further supporting this interpretation, mice infected with the mild parasite *Plasmodium chabaudi adami* also transiently demonstrated behavioural defects, generalized microglial activation, and decreased neurogenesis despite the absence of gross cerebral vascular leakage [48]. Additionally, *P. chabaudi adami*-infected animals displayed elevated levels of pro-inflammatory cytokines in brain tissue during the peak of infection (day 9 post-infection), which resolved during the recovery phase (day 15 post-infection) [48]. These data, together with the present study, suggest that while relatively mild levels of inflammation may result in transiently altered behaviour, a certain threshold is required to enact irreparable damage and eventual death.

## Conclusions

Infection of IL-10^−/−^ mice with *P. chabaudi* induces a fatal malarial disease characterized by increased mortality, severe neurological and behavioural deficits, elevated numbers of IFN-γ^+^ T cells and macrophages adherent within the brain vasculature, and activated microglia, suggestive of elevated neuroinflammation. This study adds to the growing knowledge suggesting an integral role of inflammation in neuronal damage and downstream behavioural effects observed in malaria infection. These findings contribute to the current understanding of the aetiologies of cerebral pathology and neurocognitive deficiencies in malaria infection.

## Additional file

10.1186/s12936-016-1477-1 Female IL-10^−/−^ animals succumb to *Plasmodium chabaudi* infection and exhibit increased malaria-related pathology. **A** Survival of female and male IL-10^−/−^ mice (KO) and female C57Bl/6 J mice (WT) inoculated with 10^5^ Pcc-iRBCs i.p. and followed for 16 days during the acute phase of infection. Statistical significance determined by Log-rank (Mantel-Cox) Test. **B** Animal weights (in grams) were measured during the peak of infection via digital scale. Percent change in weight determined as compared to baseline measurement (2 days pre-inoculation). Student’s t test ***p < 0.001. **C** Infection-matched female IL-10^−/−^ and WT animals were implanted with subdermal temperature-transmitting microchips and monitored daily throughout the peak of infection. Student’s t test **p < 0.01, ***p < 0.001. **D** Slides of thin blood smears from the tail vein were collected and stained to measure peripheral parasitaemia. Data points and error bars represent mean values and ± SEM, respectively.

## Abbreviations

CM: cerebral malaria
IL-10^−/−^: IL-10-deficient
WT: wild-type, C57Bl/6J
SHIRPA: SmithKline Beecham, Harwell, Imperial College, Royal London Hospital, phenotype assessment
ECM: experimental cerebral malaria
iRBCs: infected red blood cells
i.p.: intraperitoneal
ICAM-1: intracellular adhesion molecule-1
IFN-γ: interferon gamma
TNF: tumour necrosis factor
MHC-II: major histocompatibility complex class II
CXCR3: C-X chemokine receptor 3
CNS: central nervous system
PBS: phosphate-buffered saline
SEM: standard error of the mean
SD: standard deviation
FBS: fetal bovine serum
ANOVA: analysis of variance
PMA: phorbol 12-myristate 13-acetate
