# Supplementary figures and images for: Behavioural and neurological symptoms accompanied by cellular neuroinflammation in IL-10-deficient mice infected with Plasmodium chabaudi
Source: Malar J. 2016 Aug 24;15(1):428. doi: 10.1186/s12936-016-1477-1 (PMC4995805; doi:10.1186/s12936-016-1477-1)

**A**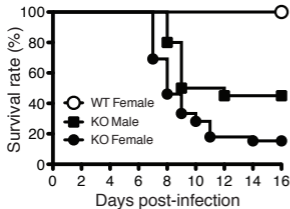**B**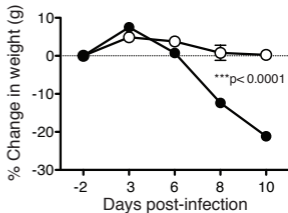**C**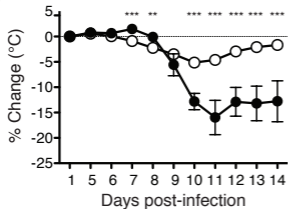**D**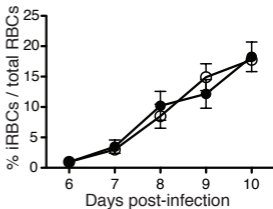

Supplement: Supplementary file 1 — 10.1186/s12936-016-1477-1 Female IL-10−/− animals succumb to Plasmodium chabaudi infection and exhibit increased malaria-related pathology. A Survival of female and male IL-10−/− mice (KO) and female C57Bl/6 J mice (WT) inoculated with 105 Pcc-iRBCs i.p. and followed for 16 days during the acute phase of infection. Statistical significance determined by Log-rank (Mantel-Cox) Test. B Animal weights (in grams) were measured during the peak of infection via digital scale. Percent change in weight determined as compared to baseline measurement (2 days pre-inoculation). Student’s t test ***p < 0.001. C Infection-matched female IL-10−/− and WT animals were implanted with subdermal temperature-transmitting microchips and monitored daily throughout the peak of infection. Student’s t test **p < 0.01, ***p < 0.001. D Slides of thin blood smears from the tail vein were collected and stained to measure peripheral parasitaemia. Data points and error bars represent mean values and ± SEM, respectively. [file 12936_2016_1477_MOESM1_ESM.pdf]
